# Crossing Virtual Doors: A New Method to Study Gait Impairments and Freezing of Gait in Parkinson's Disease

**DOI:** 10.1155/2018/2957427

**Published:** 2018-08-09

**Authors:** Luis I. Gómez-Jordana, James Stafford, C. (Lieke) E. Peper, Cathy M. Craig

**Affiliations:** ^1^Department of Human Movement Sciences, Faculty of Behavioural and Movement Sciences, Vrije Universiteit Amsterdam, Amsterdam Movement Sciences, Amsterdam, Netherlands; ^2^School of Psychology, Queens University Belfast, David Kier Building, 18-30 Malone Road, Belfast BT7 1NN, UK; ^3^INCISIV Ltd., Ormeu Avenue, Belfast, UK

## Abstract

Studying freezing of gait (FOG) in the lab has proven problematic. This has primarily been due to the difficulty in designing experimental setups that maintain high levels of ecological validity whilst also permitting sufficient levels of experimental control. To help overcome these challenges, we have developed a virtual reality (VR) environment with virtual doorways, a situation known to illicit FOG in real life. To examine the validity of this VR environment, an experiment was conducted, and the results were compared to a previous “real-world” experiment. A group of healthy controls (*N* = 10) and a group of idiopathic Parkinson disease (PD) patients without any FOG episodes (*N* = 6) and with a history of freezing (PD-f, *N* = 4) walked under three different virtual conditions (no door, narrow doorway (100% of shoulder width) and standard doorway (125% of shoulder width)). The results were similar to those obtained in the real-world setting. Virtual doorways reduced step length and velocity while increasing general gait variability. The PD-f group always walked slower, with a smaller step length, and showed the largest increases in gait variability. The narrow doorway induced FOG in 66% of the trials, while the standard doorway caused FOG in 29% of the trials. Our results closely mirrored those obtained with real doors. In short, this methodology provides a safe, personalized yet adequately controlled means to examine FOG in Parkinson's patients, along with possible interventions.

## 1. Introduction

Parkinson's disease (PD) is a degenerative disease that is characterized, in part, by the loss of dopamine-generating cells in the basal ganglia [1]. This lack of dopamine can cause bradykinesia (movement slowness), hypokinesia (reduced movement amplitude), akinesia (problems initiating movement), tremor, rigidity, and postural instability [2–4]. Approximately half of the patients with advanced stages of PD experience freezing of gait (FOG; [5]), a symptom where walking is interrupted by a brief, episodic absence, or marked reduction, of forward progression despite the intention to continue walking [3]. This debilitating symptom severely impairs mobility, hampers independence, and increases the risk of falling [3, 4].

Although the causes of FOG episodes are multifaceted, they often occur in response to certain environmental triggers (e.g., doorways) that may or may not require some kind of gait adaptation (e.g., turning or slowing down [6–10]). In this study, we aim to show how immersive, interactive virtual reality (VR) technology can offer a new methodological framework for studying FOG in people with Parkinson's. We will examine how well this technology can allow us to manipulate the visual context to induce changes in gait characteristics in PD patients who have and have not experienced FOG episodes. Moreover, the use of an immersive, interactive VR environment can allow us to determine the extent to which different visual scenes (e.g., doorways) can induce FOG and impact gait performance.

Previous FOG-inducing protocols with a high level of experimental control are very different to how PD patients normally experience episodes of FOG in real life (e.g., [11, 12]) and as a result have low ecological validity [13]. In contrast, protocols that examine FOG in ecologically valid situations (e.g., in the patient's home [14]) usually suffer from limited experimental control, compromising analytic rigor and the subsequent interpretation of the data. A notable exception to this is a study by Cowie et al. [15, 16], who asked their participants to cross real doorways constructed and presented in a laboratory setting and that were scaled to an individual participant's shoulder width. This paradigm not only induced episodes of FOG but ensured that a high level of ecological validity and experimental control was maintained. In this study, we will attempt to recreate this real-doorway paradigm in an immersive, interactive virtual reality environment that not only preserves ecological validity [17] but offers enhanced possibilities for experimental manipulation and control. Moreover, this technology also allows for the quick and personalized manipulation of the information delivered to each participant [18], yet offering reproducibility across trials.

The objective of this study was to see if a virtual environment with virtual doorways can induce FOG episodes in Parkinson's patients in the same way as real doorways [15, 16]. To examine the effectiveness of the virtual doorway manipulation, we followed Cowie et al.'s protocol [15, 16] and presented both narrow and standard doorways, comparing gait characteristics of PD patients with and without a history of FOG to those of healthy controls. We expected to find similar results to Cowie et al. [15, 16], namely, reduced step velocity, step length, and increased gait variability in Parkinson's patients, with the effects being larger for patients who have a history of FOG. It is also predicted that the FOG group will experience more freezing episodes in conditions with narrow doorways.

## 2. Methods

### 2.1. Participants

Three groups of participants were recruited: one group of healthy controls (HC; *N* = 10; mean age = 63.0 yr.; SD = 8.6 yr; 6 females), one group of idiopathic PD patients (PD; *N* = 6; mean age = 62.7 yr.; SD = 8.9 yr; 3 females), and a group of PD patients all of whom had a history of FOG (PD-f; *N* = 4; mean age = 67.5 yr.; SD = 7.0; 2 females). Motor disability was assessed using part III of the MDS-UPDRS questionnaire [19]. “The freezing of gait questionnaire” (FOGQ [20]) was used to assess if participants had experienced FOG consistently in the week prior to the experiment. A mean score >2 indicated that this was the case. The results of these tests are presented in Table 1, along with demographic information about the PD participants. The study was approved by the university's ethics committee.

### 2.2. Immersive, Interactive Virtual Reality

**Figure 1:**
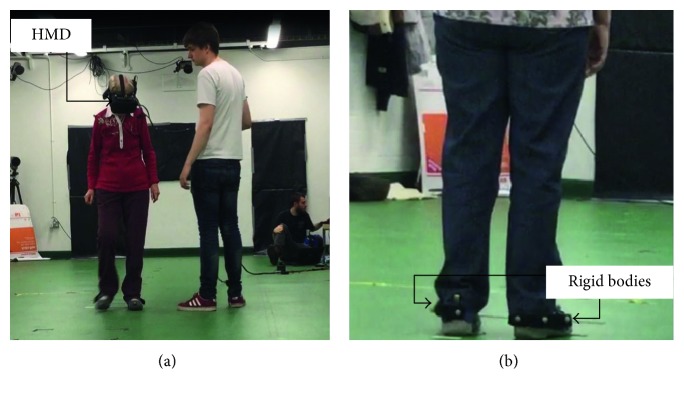
(a) A participant performing a trial. The participant wore a head-mounted display (HMD). Two experimenters are present, one to ensure the safety of the participant and another one to control the computer. (b) The two rigid bodies attached to the back of the shoes were used to track the position of the feet.

A virtual representation of a hallway was presented to participants using an Oculus Rift DK2 stereoscopic head-mounted display (Oculus VR, Irvine, California, USA). The screen had a resolution of 1920 × 1080, was updated 75 times per second, and had a field of view of 100°. To allow participants the freedom to walk up and down the virtual hallway, the Intersense IS900 (InterSense Inc., Bedford, Massachusetts, USA) tracking system was used instead of the Oculus tracking (see Figure 1(a) for image of a participant performing a trial). Both head position and orientation were tracked and updated in the virtual environment at 120 Hz. The tracked space was 12 m long by 5 m wide.

**Figure 2:**
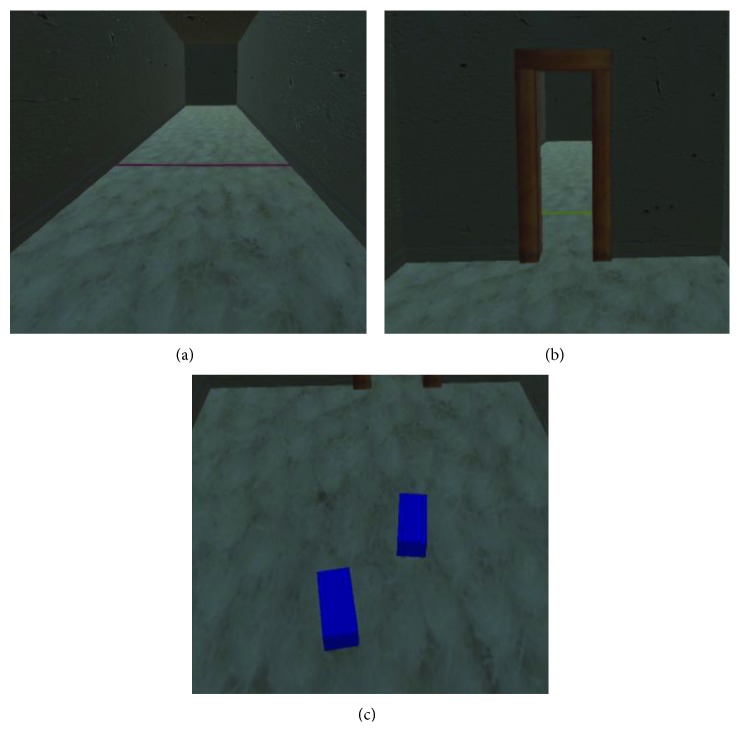
(a) The no doorway (ND) condition along with the red line indicating the end of the trial in that direction, (b) the narrow doorway (NaD) condition, and (c) a visual representation of the two cuboids that represent the placement of the participant's feet in the virtual environment (note that the image is captured from behind the participant).

The virtual environment was constructed using the games engine software Unity (version 5.4.1f1). The environment consisted of a virtual hallway 20 m long and 2.5 m high. In order to increase the realism of the environment, a clay texture was added to the walls and ceiling. Furthermore, to make the floor look as realistic as possible a texture was also added to make it look like a white carpet (Figure 2(a)).

The width of the hallway was personalized for each participant and was equivalent to 5 shoulder widths. Likewise, the width of the virtual doorways was also designed so that they were directly related to the participant's shoulder width (Figure 2(b)). Shoulder width was a parameter that was inputted at the start of each block of trials. The participants had to walk along the hallway between a red and a yellow line which were positioned 6.5 meters apart.

### 2.3. Walking Metrics

To capture gait performance data, participants had a rigid body containing three reflective markers attached to the back of each shoe (Figure 1(b)). The movement of these reflective markers was recorded at 100 Hz using 12 Qualisys infrared motion capture cameras (Qualisys Ltd., Göteborg, Sweden). In order to allow participants to see their own feet in the virtual environment, data were streamed in real-time from the infrared motion capture system into the virtual environment (Qualisys Unity SDK), 30 times a second. The position of the two rigid bodies was used to control the position and orientation of two cuboids that were used to virtually represent the position of the feet (Figure 2(c)).

### 2.4. Procedure

Before the start of the experiment, the participant's shoulder width was measured and entered into Unity to scale the width of the virtual doorway and the hallway to the participant's own bodily proportions. The experiment was carried out by two experimenters. One experimenter controlled the virtual environment and the motion capture system while the other walked next to the participant, holding the cable that connects the headset to the computer, to ensure the participant's safety. In accordance with Cowie et al. [16], three conditions were created: no virtual doorway (no door (ND)); a virtual doorway with a width that corresponded to the participant's own shoulder width (narrow door (NaD)) or to 125% of the participant's shoulder width (standard door (StaD)).(Note that the large door condition (150% shoulder width) that was included in Cowie's experiment was omitted from our study. It was found not to induce any changes in gait characteristics nor any FOG episodes in patients with an FOG history.) Supplementary Video 1 of the supplementary material includes an example of what a healthy control saw in the VR environment while completing each of the three conditions. Supplementary Video 2 includes an example of a healthy control completing one trial in each of the three conditions.

The three experimental conditions (ND, NaD, and StaD) were randomly presented six times giving a total of 18 trials. Before the start of the experiment, participants were given a familiarisation phase, where they walked up and down the virtual hallway eight times. Each time they were confronted with doors with slightly different widths to the ones used in the experimental trials.

In the conditions where a doorway was present, it was placed 4 meters in front of the participant with the red or yellow line indicating the end of the trial, positioned a further 2.5 m beyond the doorway. In all conditions, the participants were instructed to pass through the virtual doorway as they would normally pass through a doorway in real life and walk towards the yellow or red line in front of them. Once they reached the line indicating the end of the trial, the trial automatically ended and participants, with the help of the experimenter, were asked to turn around and face the opposite direction ready for the next trial. After each block of six trials, the participants were allowed to rest for 2 minutes to again minimise fatigue. The procedure lasted about 20 minutes.

### 2.5. Gait Analysis

For the conditions that involved a doorway, the gait parameter analysis (step length, cadence, and step velocity) was restricted to the area around the doorway (3.5 m before the doorway to 1 m beyond the doorway). For the no door (ND) condition, the same location was used to ensure the walking distance analysed was similar in all three conditions. The Qualisys data were analysed using custom-made Matlab (Matlab 2016b; Mathworks, Inc, Natick, Massachusetts, USA) routines. After low-pass filtering the gait data (recursive Butterworth; 2nd order; cutoff frequency: 10 Hz), heel strikes were marked automatically based on the moments at which the marker nearest to the heel reached zero velocity in a vertical direction. Step length was defined as the distance between two successive heel strikes in the walking direction. Step cadence was formalized as the number of steps taken each second. Step velocity was calculated by dividing step length by the time it took to complete that step. For each trial, the mean and coefficient of variation (CV; i.e., the ratio between the standard deviation and the mean) was calculated.

To determine whether FOG was induced in the PD-f group, we followed the analysis of Cowie et al. [16] which is based solely on step velocity. According to this method, FOG episodes were defined as sections of a trial where step velocity dropped below 10% of the mean velocity obtained for the same participant in the ND condition. If there were fewer than three strides between two identified FOG episodes, these two episodes were considered to reflect a single FOG episode. For each PD-f participant, we determined the percentage of trials in which FOG episodes were detected, as well as the mean duration of these episodes and the respective standard deviation.

### 2.6. Statistical Analysis

All statistical analyses were conducted using RStudio (RStudio 1.138; RStudio, Inc., Boston, MA). Two-way mixed ANOVAs with the between-subjects factor being group (HC, PD, and PD-f) and the within-subjects factor being door (ND, NaD, and StaD) were carried out on the means and CVs of the three gait parameters. The results were considered significant if *p* was less than 0.05. Post hoc comparisons were based on simple effects analysis [21] and (if required) pairwise *t*-tests with Bonferroni correction were used. The size of the effect was also presented using the *ω*_p_^2^ that is believed to be a better estimate of the size of the effect than *η*_p_^2^ [22]. This statistic can take values from 0 to 1, higher values indicating a higher effect size.

Kruskal–Wallis tests with a within-subject factor door (ND, NaD, and StaD) were conducted on the percentage of FOG episodes in the PD-f group. The results were considered significant if *p* was less than 0.05. If needed, post hoc Dunn tests with Bonferroni corrections were carried out to test for significant main effects. Mean durations of these episodes and respective standard deviations were not submitted for statistical analysis.

## 3. Results

### 3.1. Gait Parameters

#### 3.1.1. Mean Gait Parameter Values

**Figure 3:**
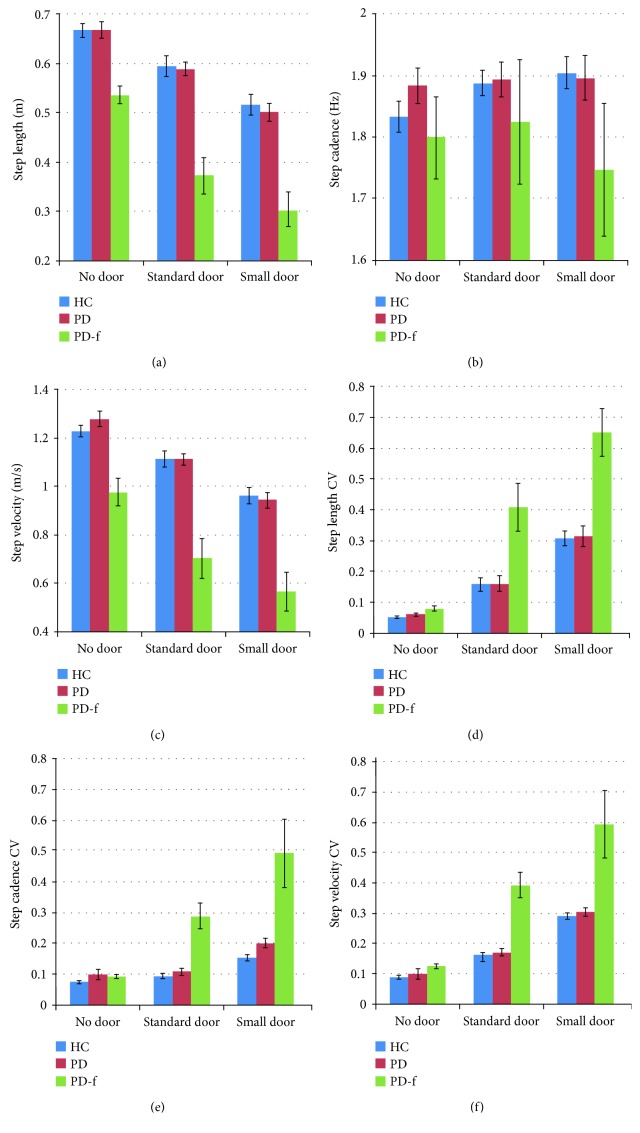
Results for step length (a), step cadence (b), step velocity (c), step length CV (d), step cadence CV (e), and step velocity CV (f) for the three groups, for the three different door conditions. The data for step length are presented in meters. The data of step cadence are presented in hertz that can be understood as the number of steps per second. Finally, the data for the step velocity are presented in meters per second (m/s). The error bars represent the standard error of the mean.

The ANOVA results for step length (Figure 3(a)) and step velocity were similar. In both cases, the main effect of door was significant (step length: *F*_(2,34)_ = 68.34, *p* < 0.001, *ω*_p_^2^ = 0.180; step velocity: *F*_(2,34)_ = 67.45, *p* < 0.001, *ω*_p_^2^ = 0.178). Post hoc analyses revealed a significantly smaller step length and lower step velocity in the NaD condition compared to the ND condition (Figure 3(c)). For both gait parameters, the effect of group was also significant (step length: *F*_(2,17)_ = 5.08, *p*=0.018, *ω*_p_^2^ = 0.026; step velocity: *F*_(2,17)_ = 5.51, *p*=0.014, *ω*_p_^2^ = 0.028), with post hoc analyses showing that both parameters were significantly smaller in the PD-f group than in the two other groups. For step cadence (Figure 3(b)), no significant effects were obtained.

#### 3.1.2. Variability (CV) of the Gait Parameters

The results of the three ANOVAs that were conducted on the gait parameter CVs also showed close correspondence. In all cases, main effects were obtained for door (step length CV: *F*_(2,34)_ = 36.87, *p* < 0.001, *ω*_p_^2^ = 0.105; step cadence: *F*_(2,34)_ = 10.20, *p* < 0.001, *ω*_p_^2^ = 0.044; step velocity: *F*_(2,34)_ = 34.51 *p* < 0.001, *ω*_p_^2^ = 0.099) and group (*F*_(2,17)_ = 4.07, *p*=0.036, *ω*_p_^2^ = 0.020; *F*_(2,17)_ = 5.65, *p*=0.013, *ω*_p_^2^ = 0.029; *F*_(2,17)_ = 5.26 *p*=0.017, *ω*_p_^2^ = 0.028, resp.),while the interaction between these two factors was also significant (*F*_(4,37)_ = 3.08, *p*=0.029, *ω*_p_^2^ = 0.015; *F*_(4,37)_ = 3.48, *p*=0.017; *ω*_p_^2^ = 0.017; *F*_(4,37)_ = 3.13, *p*=0.027; *ω*_p_^2^ = 0.015, resp.). For step length CV (Figure 3(d)) and step velocity CV (Figure 3(f)), post hoc analysis of the effect of door revealed that variability was larger in the NaD condition compared to the other two conditions. For step cadence CV (Figure 3(e)), variability in the NaD condition was larger than in the ND condition. The group effect was similar for all three CVs, showing higher variability for the PD-f group than for the other two groups. The door *x* group interaction was also similar for all three CVs, indicating that in the PD-f group, all door conditions differed significantly from one another, while in the other two groups the difference was significant only between the NaD and ND conditions. The analysis indicated that there was no significant difference between groups in the ND condition, but the difference between the PD-f group and the other groups was significant for both the NaD and the StaD doorways.

### 3.2. Freezing Episodes

Table 2 presents, per condition, the percentage of trials in which FOG episodes were detected for the individual participants who made up the PD-f group, along with information regarding the durations of these FOG episodes. The NaD condition induced at least one FOG episode in all four patients, with FOG occurring in all of the NaD trials for two of the participants (PD-f1 and PD-f4). The StaD condition was less effective in this regard, but still induced FOG in 83% of the trials in PD-f4 and 33% of the trials in PD-f1. The Kruskal–Wallis test yielded a significant effect for the factor door (*χ* ^2^ = 24.32 *p* < 0.001). Post hoc analysis revealed that the three door conditions were significantly different, with the NaD condition (66%) inducing more FOG episodes than the StaD condition (29%) which in turn caused more FOG episodes than the ND condition (4%). Supplementary Video 3 shows PD-f1 completing a trial in each doorway condition and illustrates how both doorway conditions cause her to experience a FOG episode.

## 4. Discussion

We developed an immersive, interactive VR environment that was used to manipulate the visual context within which participants control their walking along a virtual hallway. The technology not only allows us to systematically examine how environmental changes such as the presence of virtual doorways of different widths influences various gait characteristics, but also how these visual changes influence the number of FOG episodes participants with Parkinson's disease experience. By using the power of VR to recreate real-life situations commonly known to induce FOG (namely narrow doorways), we were able to measure the effects these doorways had on a participant's gait. Overall, the results obtained in our VR environment were very similar to those found in the studies conducted by Almeida and Lebold [23] and Cowie et al. [15] who used real-life stimuli. In sum, the PD-f group walked slower with smaller steps and demonstrated a higher degree of gait variability than the other two groups. When confronted with narrow doorways, all groups reduced speed and step length, compared to the condition in which no doorway was presented.

A further validation of our method was the fact that the narrow doorway elicited higher degrees of gait variability for *all* groups of participants compared to the condition without a door. Moreover, our virtual doorways induced freezing-like episodes in the majority of our PD-f patients. These episodes ranged from 1 s to 18 s, with a median of around 8 s. All of these participants had more freezing episodes when presented with the narrow doorway compared to the standard doorway. These findings clearly demonstrate how the visual information presented in the virtual environment influences gait in the same way as real doorways [16].

The close correspondence between our current results and those obtained in experiments using real doorways, speaks to the high levels of behavioural realism and ecological validity induced by the VR environment. What is interesting about this technology is that it can be easily used to examine gait in Parkinson's disease but also be used to successfully induce FOG episodes in a patient population. Moreover, VR environments can not only be used to assess the symptoms of PD, but also to test the effects potential interventions may have on improving quality of life in a safe and systematic way. For instance, the effectiveness of specific forms of visual cueing [24], to reduce FOG and improve Parkinsonian gait, may be readily examined in a VR environment. This also holds true for testing visual cueing techniques that use augmented reality glasses [25–27] which may ultimately offer a more practical application for adding virtual cues to real-life conditions. Other lines of research may also focus on the development of ecologically valid, immersive VR environments that are representative of other problematic situations for PD patients (like initiating gait or turning) and test potential intervention strategies that can be personalized to meet the needs of the individual participant.

In conclusion, immersive, interactive virtual reality is an exciting methodology that allows for the preservation of the perception/action loop. By controlling the presentation of sensory information (e.g., visual context), we can systematically and accurately measure the effects on different movement behaviours.

## Figures and Tables

**Table 1:** Demographic information about the PD participants.

Participant	Age (years)	Gender	Years from diagnosis	Clinical state	UPDRS part III	FOG-Q
PD1	61	Female	7	On	10	0.33 (0.51)
PD2	77	Male	6	Off	29	0.66 (1.06)
PD3	64	Female	2	On	18	0.33 (0.52)
PD4	66	Female	4	On	37	0.5 (0.55)
PD5	50	Male	2	On	23	0.66 (1.03)
PD6	58	Male	7	On	27	0.66(1.03)
PD-f1	68	Female	6	Off	35	3.00 (0.63)
PD-f2	76	Male	5	On	28	3.16 (0.43)
PD-f3	59	Female	4	On	36	2.66 (0.81)
PD-f4	69	Male	6	Off	41	3.33 (0.51)

The clinical state is related to the effect of the medication: on = responding well to medication; off = not responding to medication. The results of the FOGQ are presented as the mean (SD) of all the results in the test. Participants PD1-6 = idiopathic PD group; PD-f1–f4 = PD participants that have experienced freezing in the past.

**Table 2:** Characteristics of induced FOG episodes for each participant in the PD-f group as a function of door condition.

Participants	Door condition	% trials with an FOG episode	Mean duration of an FOG episode	Max duration of an FOG episode	Min duration of an FOG episode
PD-f1	No door	0	NA	NA	NA
	Standard door	33	5.64 s ± 4.07 s	8.972 s	0.51 s
	Narrow door	100	7.64 s ± 5.26 s	17.93 s	1.00 s

PD-f2	No door	0	NA	NA	NA
	Standard door	0	NA	NA	NA
	Narrow door	16	1.00 s ± 0.00 s	1.00 s	1.00 s

PD-f3	No door	0	NA	NA	NA
	Standard door	0	NA	NA	NA
	Narrow door	50	0.61 s ± 0.23 s	0.87 s	0.45 s

PD-f4	No door	16	0.85 s ± 0.00 s	0.85 s	0.85 s
	Standard door	83	2.85 s ± 3.71 s	9.45 s	0.85 s
	Narrow door	100	3.1 s ± 1.68 s	6.35 s	1.97 s

The results include percentage of trials in which patients experienced FOG; mean duration of these episodes (including standard deviation); and maximum and minimum duration of the episodes. NA indicates that no FOG episode was observed.

## Data Availability

The underlying data are available for anyone who asks for it. Please send an email to luis.jordana.martin@gmail.com.

## Supplementary Materials

Supplementary 1 Video 1 shows what the participants saw in the VR environment while they completed one trial of each of the three conditions of the experiment.

Supplementary 2 Video 2 shows a healthy participant performing one trial of each condition.

Supplementary 3 Video 3 contains a video of a PD participant performing one trial of each condition. The body of the article explicitly states when should the videos be watched.
